# Supplementary material for: The Prognostic Value of Tumor Fibrosis in Patients Undergoing Hepatic Metastasectomy for Colorectal Cancer: A Retrospective Pooled Analysis
Source: Cancers (Basel). 2025 Jun 3;17(11):1870. doi: 10.3390/cancers17111870 (PMC12153617; doi:10.3390/cancers17111870)
Supplement: Supplementary file 1 [file cancers-17-01870-s001.zip › Table S1.pdf]

Table S1. Supplementary. Hepatic Surgery Characteristics and Related Complications

	[ALL] N=108	Cohort 1 (EGFR INHIBITOR) N=54	Cohort 2 (VEGF INHIBITOR) N=54	p-value
**TIME FROM NEOADJUVANT THERAPY TO LIVER SURGERY (weeks)**	5.00 [4.00;8.25]	4.00 [3.00;7.00]	6.00 [4.00;9.75]	0.007
**PORTAL EMBOLIZACION/LIGATION:**				0.664
NO	79 (73.1%)	38 (70.4%)	41 (75.9%)	
YES	29 (26.9%)	16 (29.6%)	13 (24.1%)	
**TYPES OF LIVER SURGERY- 2 CATEGORIES:**				0.700
MINOR HEPATECTOMY	57 (52.8%)	30 (55.6%)	27 (50.0%)	
MAJOR HEPATECTOMY	51 (47.2%)	24 (44.4%)	27 (50.0%)	
**HEMORRHAGE DURING LIVER SURGERY (mL)**	648 [320;1028]	700 [361;1219]	619 [310;800]	0.420
**LIVER SURGERY DURATION (min)**	268 [185;330]	265 [186;340]	270 [185;315]	0.940
**VASCULAR CLAMPING TIME (min)**	38.0 [18.0;81.0]	48.0 [22.0;87.0]	31.5 [14.5;71.2]	0.095
**BILIARY FISTULA:**				0.489
NO	99 (91.7%)	48 (88.9%)	51 (94.4%)	
YES	9 (8.33%)	6 (11.1%)	3 (5.56%)	
**ABSCESS:**				0.266
NO	93 (86.1%)	49 (90.7%)	44 (81.5%)	
YES	15 (13.9%)	5 (9.26%)	10 (18.5%)	
**SURGICAL WOUND INFECTION:**				0.740
NO	98 (90.7%)	50 (92.6%)	48 (88.9%)	
YES	10 (9.26%)	4 (7.41%)	6 (11.1%)	
**PNEUMONIA:**				0.363
NO	103 (95.4%)	50 (92.6%)	53 (98.1%)	
YES	5 (4.63%)	4 (7.41%)	1 (1.85%)	
**HEMOPERITONEUM:**				0.032
NO	96 (88.9%)	44 (81.5%)	52 (96.3%)	
YES	12 (11.1%)	10 (18.5%)	2 (3.70%)	
**INSUFFICIENCY OR LIVER FAILURE:**				1.000
NO	93 (86.1%)	46 (85.2%)	47 (87.0%)	
YES	15 (13.9%)	8 (14.8%)	7 (13.0%)	
**ASCITES:**				0.095
NO	93 (86.1%)	43 (79.6%)	50 (92.6%)	
YES	15 (13.9%)	11 (20.4%)	4 (7.41%)	

Table S1. Supplementary *(cont)*. Hepatic Surgery Characteristics and Related Complications

	[ALL] N=108	Cohort 1 (EGFR INHIBITOR) N=54	Cohort 2 (VEGF INHIBITOR) N=54	p-value
**CLINICAL PORTAL HYPERTENSION SIGNS:**				0.158
NO	85 (78.7%)	39 (72.2%)	46 (85.2%)	
YES	23 (21.3%)	15 (27.8%)	8 (14.8%)	
**SURGICAL REINTERVENTION:**				1.000
NO	99 (91.7%)	49 (90.7%)	50 (92.6%)	
YES	9 (8.33%)	5 (9.26%)	4 (7.41%)	
**HOSPITAL STAY (days)**	7.00 [4.00;9.00]	6.00 [4.00;9.00]	7.00 [5.00;9.00]	0.619
**READMISSION:**				1.000
NO	91 (84.3%)	46 (85.2%)	45 (83.3%)	
YES	17 (15.7%)	8 (14.8%)	9 (16.7%)	
**DINDO-CLAVIEN CLASSIFICATION:**				0.036
0	46 (42.6%)	23 (42.6%)	23 (42.6%)	
1	26 (24.1%)	8 (14.8%)	18 (33.3%)	
2	8 (7.41%)	6 (11.1%)	2 (3.70%)	
3A	12 (11.1%)	5 (9.26%)	7 (13.0%)	
3B	6 (5.56%)	3 (5.56%)	3 (5.56%)	
4A	1 (0.93%)	1 (1.85%)	0 (0.00%)	
5	9 (8.33%)	8 (14.8%)	1 (1.85%)	
**DINDO-CLAVIEN - 2 CATEGORIES:**				0.272
DINDO-CLAVIEN 0-2	80 (74.1%)	37 (68.5%)	43 (79.6%)	
DINDO-CLAVIEN 3-5	28 (25.9%)	17 (31.5%)	11 (20.4%)	
**90 DAYS MORTALITY:**				0.003
NO	99 (91.7%)	45 (83.3%)	54 (100%)	
YES	9 (8.33%)	9 (16.7%)	0 (0.00%)	
