# Supplementary material for: The Prognostic Value of Tumor Fibrosis in Patients Undergoing Hepatic Metastasectomy for Colorectal Cancer: A Retrospective Pooled Analysis
Source: Cancers (Basel). 2025 Jun 3;17(11):1870. doi: 10.3390/cancers17111870 (PMC12153617; doi:10.3390/cancers17111870)
Supplement: Supplementary file 1 [file cancers-17-01870-s001.zip › Table S2.pdf]

Table S2. Supplementary. Recurrence, Postoperative Treatment, and Post-Progression Outcomes				
	**[ALL] N=108**	**Cohort 1 (EGFR INHIBITOR) N=54**	**Cohort 2 (VEGF INHIBITOR) N=54**	**p-value**
**LIVER RECURRENCE:**				1.000
NO	58 (53.7%)	29 (53.7%)	29 (53.7%)	
YES	50 (46.3%)	25 (46.3%)	25 (46.3%)	
**LUNG RECURRENCE:**				0.113
NO	67 (62.0%)	38 (70.4%)	29 (53.7%)	
YES	41 (38.0%)	16 (29.6%)	25 (46.3%)	
**NUMBER OF CHEMOTHERAPY LINES AFTER LIVER SURGERY**	2.00 [1.00;3.00]	2.00 [1.50;3.00]	2.00 [1.00;3.00]	0.246
**NUMBER OF CHEMOTHERAPY CYCLES AFTER LIVER SURGERY**	19.0 [9.00;30.0]	21.0 [9.00;28.0]	17.0 [9.00;36.2]	0.973
**NUMBER OF MONOCLONAL ANTIBODY LINES AFTER LIVER SURGERY**	1.00 [1.00;2.00]	1.00 [1.00;2.00]	1.00 [1.00;2.00]	0.225
**ADJUVANT CHEMOTHERAPY TO LIVER SURGERY OR 1st LINE mCRC[†]:**				<0.001
ONLY CHEMOTHERAPY (QT)	20 (21.5%)	8 (17.4%)	12 (25.5%)	
QT + EGFR inhibitor	28 (30.1%)	25 (54.3%)	3 (6.38%)	
QT + VEGF inhibitor	33 (35.5%)	2 (4.35%)	31 (66.0%)	
NO QT due to surgical complications	12 (12.9%)	11 (23.9%)	1 (2.13%)	
**2ond LINE CHEMOTHERAPY mCRC or 1st LINE if previous "adjuvant":**				0.010
ONLY CHEMOTHERAPY (QT)	21 (37.5%)	8 (30.8%)	13 (43.3%)	
QT + EGFR inhibitor	17 (30.4%)	13 (50.0%)	4 (13.3%)	
QT + VEGF inhibitor	18 (32.1%)	5 (19.2%)	13 (43.3%)	
**3rd LINE CHEMOTHERAPY mCRC or 2ond LINE if first "adjuvant":**				0.297
ONLY CHEMOTHERAPY (QT)	12 (42.9%)	8 (53.3%)	4 (30.8%)	
QT + EGFR inhibitor	4 (14.3%)	3 (20.0%)	1 (7.69%)	
QT + VEGF inhibitor	10 (35.7%)	4 (26.7%)	6 (46.2%)	
REGORAFENIB OR PEMBROLIZUMAB	2 (7.14%)	0 (0.00%)	2 (15.4%)	
**4th LINE CHEMOTHERAPY mCRC or 3rd LINE if first "adjuvant":**				0.127
ONLY CHEMOTHERAPY (QT)	4 (44.4%)	2 (50.0%)	2 (40.0%)	
QT + EGFR inhibitor	3 (33.3%)	0 (0.00%)	3 (60.0%)	
QT + VEGF inhibitor	2 (22.2%)	2 (50.0%)	0 (0.00%)	
**5th LINE CHEMOTHERAPY mCRC or 4th LINE if first "adjuvant":**				0.200
ONLY CHEMOTHERAPY (QT)	1 (20.0%)	0 (0.00%)	1 (33.3%)	
QT + EGFR inhibitor	2 (40.0%)	2 (100%)	0 (0.00%)	
QT + VEGF inhibitor	2 (40.0%)	0 (0.00%)	2 (66.7%)	

Table S2. Supplementary *(cont)*. Recurrence, Postoperative Treatment, and Post-Progression Outcomes

	[ALL] N=108	Cohort 1 (EGFR INHIBITOR) N=54	Cohort 2 (VEGF INHIBITOR) N=54	p-value
PROGRESSION FREE SURVIVAL FROM ADJUVANT CHEMOTHERAPY OR 1st LINE mCRC (months)	12.5 [7.00;24.0]	12.0 [7.00;23.0]	13.0 [7.00;24.0]	0.901
PROGRESSION FREE SURVIVAL FROM 2ond CHEMOTHERAPY mCRC (months)	11.5 [7.75;18.2]	10.0 [6.50;17.0]	15.0 [10.0;18.0]	0.333
PROGRESSION FREE SURVIVAL FROM 3rd CHEMOTHERAPY mCRC (months)	6.00 [4.00;9.00]	7.50 [5.25;9.50]	6.00 [4.00;8.00]	0.712
PROGRESSION FREE SURVIVAL FROM 4th CHEMOTHERAPY mCRC (months)	11.0 [10.0;12.0]	9.50 [6.25;12.8]	11.0 [10.5;11.5]	1.000
PROGRESSION FREE SURVIVAL FROM 5th CHEMOTHERAPY mCRC (months)	13.0 [12.0;16.0]	17.0 [16.5;17.5]	12.0 [6.50;12.5]	0.083

† metastatic Colorectal Cancer
