# Supplementary material for: The Prognostic Value of Tumor Fibrosis in Patients Undergoing Hepatic Metastasectomy for Colorectal Cancer: A Retrospective Pooled Analysis
Source: Cancers (Basel). 2025 Jun 3;17(11):1870. doi: 10.3390/cancers17111870 (PMC12153617; doi:10.3390/cancers17111870)
Supplement: Supplementary file 1 [file cancers-17-01870-s001.zip › Table S3.pdf]

Table S3. Supplementary. Hepatic Toxicity from Preoperative Treatment

	[ALL] N=108	Cohort 1 (EGFR INHIBITOR) N=54	Cohort 2 (VEGF INHIBITOR) N=54	p-value
**TOXICITY ON THE HEPATIC SINUSOID:**				0.008
NONE	32 (29.6%)	15 (27.8%)	17 (31.5%)	
MILD	50 (46.3%)	19 (35.2%)	31 (57.4%)	
MODERATE	23 (21.3%)	17 (31.5%)	6 (11.1%)	
SEVERAL	3 (2.78%)	3 (5.56%)	0 (0.00%)	
**TOXICITY ON THE HEPATIC SINUSOID - 2 CATEGORIES:**				0.003
NONE-MILD	82 (75.9%)	34 (63.0%)	48 (88.9%)	
MODERATE-SEVERAL	26 (24.1%)	20 (37.0%)	6 (11.1%)	
**LIVER STEATOSIS:**				0.071
< 30%	86 (85.1%)	38 (77.6%)	48 (92.3%)	
≥ 30%	15 (14.9%)	11 (22.4%)	4 (7.69%)	
**NON-ALCOHOLIC STEATOHEPATITIS (NASH):**				0.330
< 5%	47 (94.0%)	26 (92.9%)	21 (95.5%)	
5-33%	2 (4.00%)	2 (7.14%)	0 (0.00%)	
>33-66%	1 (2.00%)	0 (0.00%)	1 (4.55%)	
**CLINICAL PORTAL HYPERTENSION SIGNS:**				0.158
NO	85 (78.7%)	39 (72.2%)	46 (85.2%)	
YES	23 (21.3%)	15 (27.8%)	8 (14.8%)	
