# Supplementary material for: The Prognostic Value of Tumor Fibrosis in Patients Undergoing Hepatic Metastasectomy for Colorectal Cancer: A Retrospective Pooled Analysis
Source: Cancers (Basel). 2025 Jun 3;17(11):1870. doi: 10.3390/cancers17111870 (PMC12153617; doi:10.3390/cancers17111870)
Supplement: Supplementary file 1 [file cancers-17-01870-s001.zip › Table S4.pdf]

Table S4. Supplementary. UNIVARIATE OVERALL SURVIVAL ANALYSIS-CLINICAL AND NEOPLASIA-RELATED				
**Characteristic**	**N**	**HR†**	**95% CI‡**	**p-value**
**CLINICAL AND NEOPLASIA-RELATED VARIABLES**				
**GENDER:**	99			0.033
MALE (reference)	61	1		
FEMALE	38	0.6	0.37, 0.97	
**AGE AT LIVER SURGERY**	99	1	0.99, 1.01	>0.99
**BMI* CATEGORIES:**	99			0.9
LOW OR NORMAL WEIGHT (reference)	47	1		
OVERWEIGHT OR OBESITY	52	0.97	0.62, 1.52	
**PRIMARY TUMOR LOCATION (RIGHT vs LEFT):**	99			0.13
RIGHT COLON (reference)	16	1		
LEFT COLON	83	0.63	0.36, 1.11	
**OPERATED PRIMARY TUMOR:**	99			0.009
NO (reference)	5	1		
YES	94	0.22	0.09, 0.56	
**TIME TO PRIMARY TUMOR SURGERY:**	94			0.92
BEFORE NEOADJUVANT TREATMENT (reference)	51	1		
AFTER NEOADJUVANT TREATMENT	43	0.98	0.62, 1.55	
**LYMPH NODE RATIO (LNR)**	92	3.99	1.25, 12.7	0.032
**STATUS_KRAS:**	95			0.46
WILD-TYPE (reference)	58	1		
MUTANT	37	1.19	0.75, 1.89	
**STATUS_NRAS:**	66			0.93
WILD-TYPE (reference)	64	1		
MUTANT	2	1.09	0.15, 8.02	
**RAS_BRAF_MUTATIONS:**	95			0.32
NO RAS/BRAF MUTATIONS (reference)	55	1		
RAS OR BRAF MUTATIONS	40	1.26	0.80, 2.00	
**CEA AT THE METASTATIC DIAGNOSIS (ng/mL)**	98	1	1.00, 1.00	0.42
**CA 19.9 AT THE METASTATIC DIAGNOSIS (U/mL)**	81	1	1.00, 1.00	0.79
**SYNCHRONOUS VS METACHRONOUS LIVER METASTASES:**	99			0.43
SYNCHRONOUS (reference)	79	1		
METACHRONOUS	20	0.8	0.45, 1.42	
**UNI OR BILOBAR LIVER METASTASES:**	99			0.086
UNILOBAR (reference)	52	1		
BILOBAR	47	0.67	0.43, 1.06	
**NUMBER OF LIVER METASTASES**	98	1.03	0.99, 1.09	0.17
**NUMBER OF LIVER SEGMENTS AFFECTED**	98	1.11	0.98, 1.27	0.11
**SIZE OF LIVER METASTASES ≥ 5 cm:**	94			0.16
M1 < 5 CM (reference)	51	1		
M1 ≥ 5 CM	43	1.4	0.88, 2.23	
**SIZE OF LIVER METASTASES ≥ 10 cm:**	94			0.044
M1 < 10 CM (reference)	86	1		
M1 ≥ 10 CM	12	2.34	1.11, 4.92	

Table S4. Supplementary*(cont)*. UNIVARIATE OVERALL SURVIVAL ANALYSIS-CLINICAL AND NEOPLASIA-RELATED

Characteristic	N	HR†	95% CI‡	p-value
**CLINICAL AND NEOPLASIA-RELATED VARIABLES**				
**HEPATOPATHY PRIOR TO LIVER SURGERY:**	93			<0.001
NONE OR MILD HEPATOPATHY (reference)	51	1		
MODERATE OR SEVERE HEPATOPATHY	42	2.63	1.63, 4.26	
**CLINICAL RISC SCORE (FONG *et al*) - 2 CATEGORIES**	87			0.6
CLINICAL RISK SCORE 0-2 (reference)	33	1		
CLINICAL RISK SCORE 3-5	54	1.14	0.69, 1.87	

†HR = Hazard Ratio, ‡CI = Confidence Interval

* Body Mass Index
