# Supplementary material for: The Prognostic Value of Tumor Fibrosis in Patients Undergoing Hepatic Metastasectomy for Colorectal Cancer: A Retrospective Pooled Analysis
Source: Cancers (Basel). 2025 Jun 3;17(11):1870. doi: 10.3390/cancers17111870 (PMC12153617; doi:10.3390/cancers17111870)
Supplement: Supplementary file 1 [file cancers-17-01870-s001.zip › Table S5.pdf]

Table S5. Supplementary. UNIVARIATE OVERALL SURVIVAL ANALYSIS-TREATMENT-RELATED				
**Characteristic**	**N**	**HR†**	**95% CI‡**	**p-value**
**TREATMENT-RELATED VARIABLES**				
**CHEMOTHERAPY SCHEDULE BEFORE LIVER SURGERY**	99			0.68
OXALIPLATIN + 5FU (reference)	65	1		
IRINOTECAN + 5FU	34	1.1	0.70, 1.75	
**NUMBER OF CHEMOTHERAPY CYCLES BEFORE LIVER SURGERY**	99	1	0.97, 1.04	0.86
**CUMULATIVE DOSE OF OXALIPLATIN (mg/m2)**	65	1	1.00, 1.00	0.57
**CUMULATIVE DOSE OF IRINOTECAN (mg/m2)**	34	1	1.00, 1.00	0.49
**MONOCLONAL ANTIBODY (MO-Ab) BEFORE LIVER SURGERY**	99			0.96
EGFR INHIBITOR (reference)	45	1		
VEGF INHIBITOR	54	0.99	0.63, 1.55	
**NUMBER OF MO-Ab CYCLES BEFORE LIVER SURGERY**	99	1	0.96, 1.04	0.96
**EGFR INHIBIDOR RELATED SKIN TOXICITY (CTC-AE):**	45			0.33
1 (reference)	19	1		
2	17	0.96	0.45, 2.05	
3	9	0.5	0.18, 1.41	
**CEA BEFORE LIVER SURGERY (ng/mL)**	94	1*	1.00, 1.00	0.006
**CA 19.9 BEFORE LIVER SURGERY (U/mL)**	77	1	1.00, 1.00	>0.99
**PORTAL EMBOLIZACION/LIGATION:**	99			0.52
NO (reference)	73	1		
YES	26	1.18	0.72, 1.93	
**TYPES OF LIVER SURGERY- 2 CATEGORIES**	99			0.85
MINOR HEPATECTOMY (reference)	54	1		
MAJOR HEPATECTOMY	45	0.96	0.61, 1.49	
**RESECTED PRIMARY TUMOR AND ALL METASTASES**	99			
YES (CR0) (reference)	87	1		
NO (CR2)	12	1.96	1.03, 3,74	0.04
**HAEMORRHAGE DURING LIVER SURGERY (mL)**	82	1	1.00, 1.00	0.22
**LIVER SURGERY DURATION (min)**	83	1	1.00, 1.00	0.33
**HEPATIC CLAMPING TIME (min)**	92	1	0.99, 1.00	0.63
**DINDO-CLAVIEN - 2 CATEGORIES**	99			0.081
DINDO-CLAVIEN 0-2 (reference)	79	1		
DINDO-CLAVIEN 3-5	20	1.62	0.96, 2.74	

†HR = Hazard Ratio, ‡CI = Confidence Interval

* 1.001 (CI95 1.0004-1.0024)
