# Supplementary material for: The Prognostic Value of Tumor Fibrosis in Patients Undergoing Hepatic Metastasectomy for Colorectal Cancer: A Retrospective Pooled Analysis
Source: Cancers (Basel). 2025 Jun 3;17(11):1870. doi: 10.3390/cancers17111870 (PMC12153617; doi:10.3390/cancers17111870)
Supplement: Supplementary file 1 [file cancers-17-01870-s001.zip › Table S6.pdf]

Table S6. Supplementary. UNIVARIATE OVERALL SURVIVAL ANALYSIS-RESPONSE-RELATED				
**Characteristic**	**N**	**HR†**	**95% CI‡**	**p-value**
**RESPONSE-RELATED VARIABLES**				
**PATHOLOGICAL MAJOR SIZE OF LIVER METASTASES (mm)**	90	1.01	1.00, 1.02	0.036
**RESECTION MARGIN DISTANCE (mm)**	93	0.95	0.88, 1.03	0.16
**MEDIAN RESIDUAL TUMOR (%)**	99	1.01	1.00, 1.02	0.011
**MEDIAN RESIDUAL FIBROSIS (%)**	99	0.99	0.98, 1.00	0.1
**MEDIAN RESIDUAL NECROSIS (%)**	99	1	0.99, 1.01	0.86
**PATHOLOGICAL RESPONSE BY RUBBIA-BRANDT CRITERIA - 2 CATEGORIES**	99			0.088
TRG NOT RESPONDERS (TRG4-5) (reference)	42	1		
TRG RESPONDERS (TRG1-3)	57	0.68	0.43, 1.06	
**PATHOLOGICAL RESPONSE BY POULTSIDES CRITERIA**	99			0.034
FIBROSIS < 40% (reference)	60	1		
FIBROSIS ≥ 40%	39	0.61	0.38, 0.97	
**NECROSIS CATEGORIES**	99			0.44
NECROSIS < 40% (reference)	68	1		
NECROSIS ≥ 40%	31	1.21	0.75, 1.93	
**TOXICITY ON THE HEPATIC SINUSOID - 2 CATEGORIES**	99			0.4
NONE-MILD (reference)	76	1		
MODERATE-SEVERAL	23	0.79	0.46, 1.38	
**LIVER STEATOSIS**	92			0.82
< 30% (reference)	78	1		
≥ 30%	14	1.08	0.55, 2.12	

†HR = Hazard Ratio, ‡CI = Confidence Interval
